# The lncRNA MALAT1 rs619586 G Variant Confers Decreased Susceptibility to Recurrent Miscarriage

**DOI:** 10.3389/fphys.2019.00385

**Published:** 2019-04-09

**Authors:** Di Che, Yanfang Yang, Yufen Xu, Zhenzhen Fang, Lei Pi, LanYan Fu, Huazhong Zhou, Yaqian Tan, Zhaoliang Lu, Li Li, Qihua Liang, Qingshan Xuan, Xiaoqiong Gu

**Affiliations:** ^1^Department of Clinical Biological Resource Bank, Guangzhou Institute of Pediatrics, Guangzhou Women and Children’s Medical Center, Guangzhou Medical University, Guangzhou, China; ^2^Department of Prenatal Diagnosis, Maoming People’s Hospital, Maoming, China; ^3^Program of Molecular Medicine, Guangzhou Women and Children’s Hospital, Zhongshan School of Medicine, Sun Yat-sen University, Guangzhou, China; ^4^Department of Gynecology, Guangzhou Women and Children’s Medical Center, Guangzhou Medical University, Guangzhou, China; ^5^Department of Clinical Lab, Guangzhou Women and Children’s Medical Center, Guangzhou Medical University, Guangzhou, China; ^6^Department of Blood Transfusion, Guangzhou Women and Children’s Medical Center, Guangzhou Medical University, Guangzhou, China

**Keywords:** recurrent miscarriage, MALAT1, susceptibility, rs619586, single nucleotide polymorphism

## Abstract

Cardiovascula disease and recurrent miscarriage have shared risk factors, and some cardiovascular disease-related candidate genes have been confirmed to be associated with recurrent miscarriage. Metastasis-associated lung adenocarcinoma transcript 1 (MALAT1) is a long non-coding RNA (lncRNA) that is considered to be associated with susceptibility to cardiovascular disease. However, whether lncRNA MALAT1 polymorphisms are related to recurrent miscarriage susceptibility is unclear. We genotyped three lncRNA MALAT1 polymorphisms (rs591291, rs619586, and rs3200401) in 284 patients and 392 controls using TaqMan methods. Logistic regression was used to evaluate the odds ratios (ORs) and 95% confidence intervals (CIs) adjusted for age. Our results showed that the rs619586 G variant had protective effects against recurrent miscarriage (AG vs. AA: adjusted OR = 0.670, 95% CI = 0.457–0.982, *p* = 0.040; GG vs. AA: adjusted OR = 0.278, 95% CI = 0.079–0.975, *p* = 0.046; GG/AG vs. AA adjusted OR = 0.621, 95% CI = 0.429–0.900, *p* = 0.012). In a combined analyses of protective genotypes, with regard to the three single nucleotide polymorphisms (SNPs), we found that individuals with two or three protective genotypes exhibited a significantly lower risk of recurrent miscarriage than those with no or only one protective genotype (adjusted OR = 0.369, 95% CI = 0.199–0.684, *p* = 0.002). Moreover, the decrease in recurrent miscarriage risk with two or three protective genotypes was most pronounced in women less than 35 years of age (OR = 0.290, 95% CI = 0.142–0.589, *p* < 0.001) and in women with 2–3 miscarriages (adjusted OR = 0.270, 95% CI = 0.126–0.580, *p* < 0.001). In conclusion, our study suggests that the rs619586 G variant may have potential protective effects conferring a decreased risk of recurrent miscarriage in the southern Chinese population.

## Introduction

Recurrent miscarriage is defined as the loss of two or more consecutive pregnancies before 20 weeks of gestation (Jaslow et al., 2010; Diejomaoh, 2015). The occurrence of recurrent miscarriage is associated with many factors, including genetic factors, immunological dysfunction, endocrine disorders, unhealthy lifestyles and defects of the reproductive organs (Saravelos and Regan, 2014; Sen et al., 2014; Garrido-Gimenez and Alijotas-Reig, 2015; Kaur and Gupta, 2016; Pereza et al., 2017; Shi et al., 2017). In recent years, many studies have revealed a relationship between miscarriage and cardiovascular disease. Cardiovascular disease and recurrent miscarriage share risk factors, and women who experience miscarriages may have an increased risk of cardiovascular disease (Kharazmi et al., 2010; El Achi et al., 2018). For example, women with a history of miscarriage appear to have an increased risk of ischemic heart disease (Wagner et al., 2015). Smith et al. (2011) reported that the parents of women who have experienced recurrent miscarriage also have an increased risk of ischemic heart disease, and Kharazmi et al. (2010) suggests that women who experience spontaneous pregnancy loss may have an increased risk of myocardial infarction (Zhu et al., 2018). Moreover, studies have found that genetic polymorphisms may be involved in the pathogenesis of recurrent miscarriage (Hyde and Schust, 2015), and some cardiovascular disease-related candidate genes have been confirmed to be associated with recurrent miscarriage, such as MTHFR (C677T), APO-E3, and Factor II (G20210A) (El Achi et al., 2018).

Metastasis-associated lung adenocarcinoma transcript 1 (MALAT1) is a long non-coding RNA (lncRNA) that participates in splicing and epigenetic regulation (Tripathi et al., 2010; Yang et al., 2011). Recent studies have found that the MALAT1 gene polymorphism is associated with a susceptibility to various diseases, such as cancer risk and congenital heart disease (Huang et al., 2018; Li et al., 2018). Moreover, MALAT1 is involved in angiogenesis and inflammation, where inflammation is associated with the occurrence of recurrent miscarriage, and recurrent miscarriage may increase the risk of cardiovascular disease (Thum and Fiedler, 2014; Vitagliano et al., 2017). Studies have also confirmed that the expression level of MALAT1 was reduced in the villus samples of recurrent miscarriage patients and the regulation of MALAT1 is one of the factors that contributes to the pathogenesis of recurrent miscarriage (Wang Y. et al., 2018). These studies suggest that the MALAT1 gene polymorphism may be associated with recurrent miscarriage. However, reports have not focused on whether the MALAT1 gene polymorphism is associated with miscarriage susceptibility. Research has confirmed that the rs619586 G allele of the MALAT1 gene is associated with a decreased risk of coronary atherosclerotic heart disease (Wang G. et al., 2018), and genetic variations in MALAT1 (rs591291) showed a significantly better hepatocellular cancer prognoses in female patients (Wang B.G. et al., 2018). Furthermore, the lncRNA MALAT1 rs619586 and rs3200401 variants are associated with a decreased susceptibility to breast cancer (Peng et al., 2018). Therefore, we investigated whether recurrent miscarriage susceptibility is related to specific MALAT1 gene polymorphisms (rs591291, rs619586, and rs3200401) in a case-control study that included 248 cases and 392 controls from a southern Chinese population.

## Materials and Methods

### Study Population

In the current study, a total of 248 recurrent miscarriage patients and 392 healthy controls were recruited at the Gynecology Department of Guangzhou Women and Children’s Medical Center, between June 2017 and July 2018. Recurrent miscarriage was diagnosed as the occurrence of two or more spontaneous miscarriages of unknown etiology (5–24 weeks of gestation) with the same husband, and the control women had had at least two normal pregnancies and no history of a miscarriage. None of the patients with recurrent spontaneous miscarriage or the control women had a history of metabolic disorders, autoimmune conditions, hypertension, endocrine disorders, arterial or venous thrombosis, uterine anomalies, liver or kidney dysfunction, or embryo chromosomal abnormalities. Chromosomal abnormalities were excluded in all couples in the recurrent miscarriage group.

This study was approved by the Ethics Committee of Guangzhou Women and Children’s Medical Center (201802202, Guangzhou, China). Written informed consent was obtained from each recurrent miscarriage patient and control subject before participation in the study. The clinical data, personal information, and demographic information were collected with a medical record system.

### SNP Genotyping and DNA Extraction

Genomic DNA was extracted from 200 μL samples of peripheral blood leukocytes from all participants by using a Blood DNA Isolation Kit (Tiangen, Beijing, China) according to the manufacturer’s instructions. The specific fluorescent probes for single nucleotide polymorphism (SNP) (rs591291, rs619586, and rs3200401) genotyping were purchased from ABI (Thermo Fisher Scientific, United States). Genotyping of the three SNPs was performed in a 384-well plate on an ABI Q6 instrument (Thermo Fisher Scientific, United States) according to the TaqMan real-time polymerase chain reaction protocol. A random selection of 10% of the samples was repeated for detection, and the results showed 100% concordance.

### Statistical Analysis

The data were analyzed using SAS statistical analysis software (version 9.4; SAS Institute, Cary, NC, United States). The tests were two-tailed, and *P*-values less than 0.05 were considered statistically significant. Hardy-Weinberg equilibrium (HWE) for the control group was calculated using the goodness-of-fit χ^2^ test. Odds ratios (ORs) and 95% confidence intervals (95% CIs) were used to estimate the associations between the MALAT1 gene polymorphisms (rs591291, rs619586, and rs3200401) and recurrent miscarriage susceptibility, based on extracted genotype data. Adjusted ORs were calculated using multiple-variable unconditional logistic regression after adjustment for age. In addition, analyses stratified by age and the number of miscarriages were performed. We divided the patients into two groups according to the number of abortion occurrences (two to three miscarriages or four or more miscarriages).

## Results

### Population Characteristics and SNP Selection

In total, we recruited 248 recurrent miscarriage patients and 392 healthy controls with ages ranging from 20 to 44 (Table 1). There was no significant difference between the recurrent miscarriage patients and the controls in terms of age (31.00 ± 4.83 vs. 31.44 ± 4.39 years, *p* = 0.722). Approximately 68.15% of the recurrent miscarriage patients had undergone two or three spontaneous miscarriages, and more than 31.85% had suffered four or more spontaneous miscarriages.

**Table 1:** Frequency distribution of selected characteristics of the recurrent miscarriage and control groups.

Variables	Patients (*n* = 248)		Controls (*n* = 392)		*P*^a^
		
	No.	%	No.	%	
Age range, years	20–44		22–44		0.722
Mean ± SD	31.00 ± 4.83		31.44 ± 4.39		
<35	187	75.4	288	73.47	
35–40	52	20.97	92	23.47	
>40	9	3.63	12	3.06	
**No. of abortions/%**
2–3	169	68.15			
≥4	79	31.85			


*^a^Two-sided χ^*2*^ test for distributions of recurrent miscarriage patients and controls*.

### Association Between MALAT1 Gene Polymorphisms and Recurrent Miscarriage Susceptibility

The genotype frequency distribution of the three SNPs was analyzed with the goodness-of-fit χ^2^ test. As shown in Table 2, upon analysis of the genotypic and allelic frequencies of SNPs between the recurrent miscarriage patients and the healthy controls, the *P*-values for HWE in the control group were above 0.05 (*p* = 0.227 for rs591291, *p* = 0.123 for rs3200401, and *p* = 0.259 for rs619586), suggesting that the genotype frequencies for those SNPs conformed to HWE. Single-locus analysis suggested that the rs619586 G variant in lncRNA MALAT1 was associated with decreased recurrent miscarriage susceptibility (AG vs. AA: adjusted OR = 0.670, 95% CI = 0.457–0.982, *p* = 0.040; GG vs. AA: adjusted OR = 0.278, 95% CI = 0.079–0.975, *p* = 0.046; GG/AG vs. AA adjusted OR = 0.621, 95% CI = 0.429–0.900, *p* = 0.012). However, we found no significant relationship between rs591291 or rs3200401 in lncRNA MALAT1 and recurrent miscarriage risk. Upon combined analysis of the protective genotypes with regard to the three SNPs, we found that individuals with two or three protective genotypes exhibited significantly lower recurrent miscarriage risk than those with no or only one protective genotype (adjusted OR = 0.369, 95% CI = 0.199–0.684, *p* = 0.002).

**Table 2:** Genotype and allele frequencies of MALAT1 in recurrent miscarriage patients and controls.

Genotype/allele	RM (*N* = 248)	Controls (*N* = 392)	*P*-value^a^	OR (95% CI)	*P*-value	Adjusted OR (95% CI)	*P*-value^b^	
**MALAT1/rs591291 C > T (HWE = 0.227)**
CC	84 (33.87)	149 (38.01)	0.531	1.00	/	1.00	/	
CT	116 (46.77)	176 (44.90)	/	1.169 (0.819–1.668)	0.389	1.163 (0.815–1.661)	0.405	
TT	48 (19.35)	67 (17.09)	/	1.271 (0.805–2.007)	0.304	1.271 (0.805–2.009)	0.306	
Dominant	164 (66.13)	243 (61.99)	0.288	1.197 (0.858–1.670)	0.289	1.193 (0.855–1.665)	0.299	
Recessive	200 (80.65)	325 (82.91)	0.469	1.164 (0.772–1.755)	0.468	1.168 (0.774–1.761)	0.459	
C	284 (57.26)	474 (60.46)	0.257	1.00	/	1.00		/
T	212 (42.74)	310 (39.54)		1.141 (0.908–1.434)	0.256	1.141 (0.908–1.434)		0.257
**MALAT1/rs3200401 C > T (HWE = 0.123)**
CC	180 (72.58)	277 (70.66)	0.412	1.00	/	1.00	/	
CT	63 (25.40)	100 (25.51)	/	0.969 (0.672–1.399)	0.869	0.963 (0.667–1.390)	0.840	
TT	5 (2.02)	15 (3.83)	/	0.513 (0.183–1.437)	0.204	0.507 (0.181–1.420)	0.196	
Dominant	68 (27.42)	115 (29.34)	0.601	0.910 (0.639–1.296)	0.601	0.903 (0.634–1.287)	0.573	
Recessive	243 (97.98)	377 (96.17)	0.187	0.518 (0.186–1.442)	0.208	0.512 (0.184–1.428)	0.201	
C	423 (85.28)	654 (83.42)	0.372	1.00	/	1.00		/
T	73 (14.72)	130 (16.58)		0.868 (0.636–1.186)	0.374	0.863 (0.632–1.179)		0.354
**MALAT1/rs619586 A > G (HWE = 0.259)**
AA	194 (78.23)	271 (69.13)	**0.014**	1.00	/	1.00	/	
AG	51 (20.56)	106 (27.04)	/	**0.672 (0.459–0.984)**	**0.041**	**0.670 (0.457–0.982)**	**0.040**	
GG	3 (1.21)	15 (3.83)	/	**0.279 (0.080–0.978)**	**0.046**	**0.278 (0.079–0.975)**	**0.046**	
Dominant	54 (21.77)	121 (30.87)	**0.011**	**0.623 (0.431–0.903)**	**0.012**	**0.621 (0.429–0.900)**	**0.012**	
Recessive	245 (98.79)	377 (96.17)	**0.039**	0.308 (0.088–1.074)	0.065	0.307 (0.088–1.072)	0.064	
A	439 (88.51)	648 (82.65)	**0.004**	1.00	/	1.00		/
G	57 (11.49)	136 (17.53)		**0.619 (0.444–0.862)**	**0.005**	**0.617 (0.442–0.860)**		**0.004**
**Combined protective effect of genotypes^∗^**
0–1	233 (93.95)	332 (84.69)	**<0.001**	1.00	/	1.00	/	
2–3	15 (6.05)	60 (15.31)		**0.373 (0.201–0.690)**	**0.002**	**0.369 (0.199–0.684)**	**0.002**	


*^∗^The protective genotypes used for the calculation were rs591291CC+rs3200401 CT/TT+rs619586 AG/GG. ^*a*^χ^*2*^ test for genotype distributions between recurrent miscarriage patients and controls. ^*b*^Adjusted for age. RM: recurrent miscarriage patients. Statistically significant values are shown in bold (*P* < 0.05)*.

### Stratified Analysis of Selected Polymorphisms and Recurrent Miscarriage Susceptibility

We further explored the associations between lncRNA MALAT1 gene polymorphisms (rs619586) and combined effects of protective genotypes and recurrent miscarriage susceptibility in analyses stratified by age and number of miscarriages (as shown in Table 3). Compared with the rs619586 AA variant, the AG/GG variant was more protective in women less than 35 years of age (OR = 0.534, 95% CI = 0.345–0.827, *p* = 0.005) and in women who had undergone 2–3 miscarriages (adjusted OR = 0.577, 95% CI = 0.375–0.888, *p* = 0.012). Moreover, the combined analysis suggested that the presence of two or three protective genotypes decreased the recurrent miscarriage risk in women less than 35 years of age (OR = 0.290, 95% CI = 0.142–0.589, *p* < 0.001) and in women who had undergone 2–3 miscarriages (adjusted OR = 0.270, 95% CI = 0.126–0.580, *p* < 0.001) compared with the presence of no or only one protective variant.

**Table 3:** Stratification analysis of associations between MALAT1 polymorphisms and recurrent miscarriage risk in a southern Chinese population.

Variable	rs619586 (cases/controls)		*p*	OR (95% CI)	*p*	Adjusted OR (95% CI)	*p*^a^	Combined (cases/controls)^∗^		*p*	OR (95% CI)	*p*	Adjusted OR (95% CI)	*p*^a^
	AA	AG/GG						0–1	2–3					
**Age, years**
<35	**150/197**	**37/91**	**0.004**	**0.534 (0.345–0.827)**	**0.005**	**/**	**/**	**177/241**	**10/47**	**<0.001**	**0.290 (0.142–0.589)**	**<0.001**	**/**	/
35–40	38/65	14/27	0.756	0.887 (0.415–1.896)	0.757	/	/	48/80	4/12	0.314	0.556 (0.170–1.821)	0.332	/	/
>40	6/9	3/3	0.677	1.500 (0.223–10.077)	0.677	/	/	8/11	1/1	0.831	1.375 (0.074–25.433)	0.831	/	/
**No. of abortions/%**			
2–3	134/271	35/121	**0.012**	**0.585 (0.381–0.381)**	**0.014**	**0.577 (0.375–0.888)**	**0.012**	161/332	8/60	**<0.001**	**0.275 (0.128–0.589)**	**<0.001**	**0.270 (0.126–0.580)**	**<0.001**
4≥	60/271	19/121	0.219	0.709 (0.406–1.240)	0.228	0.723 (0.413–1.266)	0.256	72/332	7/60	0.116	0.538 (0.236–1.225)	0.140	0.549 (0.241–1.253)	0.155


*^∗^The combination of protective genotypes used for the calculation were MALAT1: rs591291CC+rs3200401 CT/TT+rs619586 AG/GG. ^*a*^Adjusted for age. RM, recurrent miscarriage patients. Statistically significant values are shown in bold (*P* < 0.05)*.

### FPRP Values for All Significant Associations

In Table 4, the false-positive report probability (FPRP) values of the positive results of the MALAT1 gene discovery are shown. The predicted value of the false positive report was 0.2, and the prior probability was 0.1. Compared with the rs619586 A genotype carrier, the probability that the rs619586 G genotype can reduce the risk of recurrent abortion is still credible (FPRP = 0.115). In the FPRP analysis, most of the meaningful findings are not noteworthy, which is likely due to the limited sample size in the current study. Therefore, important findings from current research need to be further validated with large sample sizes.

**Table 4:** False-positive report probability values for associations between recurrent miscarriage risk and genotypes of MALAT1 polymorphisms.

Genotype/allele	OR (95% CI)	*P*-value^a^	Statistical power^b^	Prior probability				
				0.25	0.1	0.01	0.001	0.0001
**MALAT1/rs619586 A > G**
AG vs. AA	0.672 (0.459–0.984)	0.041	0.505	**0.196**	0.422	0.889	0.988	0.999
GG vs. AA	0.279 (0.080–0.978)	0.046	0.115	0.546	0.783	0.975	0.998	1.000
AG/GG vs. AA	0.623 (0.431–0.903)	0.012	0.358	**0.091**	0.232	0.769	0.971	0.997
G vs. A	0.619 (0.444–0.862)	0.005	0.346	**0.042**	**0.115**	0.589	0.935	0.993
**AG/GG vs. AA**
<35 years	0.534 (0.345–0.827)	0.005	0.171	**0.081**	0.208	0.743	0.967	0.997
2–3 abortions	0.585 (0.381–0.381)	0.014	0.277	**0.132**	0.313	0.834	0.981	0.998
**Protective genotypes**							
0–1 vs. 2–3	0.373 (0.201–0.690)	0.002	0.064	**0.086**	0.219	0.755	0.969	0.997
<35 years	0.290 (0.142–0.589)	0.001	0.028	**0.096**	0.243	0.779	0.973	0.997
2–3 abortions	0.275 (0.128–0.589)	0.001	0.023	**0.115**	0.281	0.811	0.977	0.998


*^a^Calculated the genotype frequency distributions using the omnibus χ^*2*^ test in Tables 2, 3. ^*b*^Calculated the statistical power using the number of observations and the OR and *P*-values in Tables 2, 3. Statistically significant values are shown in bold (*P* < 0.05).*

## Discussion

To the best of our knowledge, this study is the first to investigate the associations between lncRNA MALAT1 gene polymorphisms (rs619586, rs3200401, and rs591291) and recurrent miscarriage susceptibility. Included in our study were 248 recurrent miscarriage patients and 392 healthy controls. Our results suggested that the lncRNA MALAT1 rs619586 G allele was associated with a decreased risk of recurrent miscarriage, and the protective effect was most pronounced in women less than 35 years of age, and in the subgroup of women with two to three prior miscarriages. In contrast, other lncRNA MALAT1 SNPs (rs3200401 and rs591291) were not associated with recurrent miscarriage susceptibility.

MALAT1 is one of the lncRNAs that has been proven to be associated with disease, and a growing number of studies have indicated that MALAT1 also participates in various pathological processes (Wu et al., 2015). Several studies have revealed that MALAT1 gene polymorphisms are associated with disease susceptibility. For example, the MALAT1 rs619586 G variant was associated with a decreased risk of hepatocellular carcinoma and colorectal cancer (Liu et al., 2012; Zhao et al., 2018). In addition, Wang G. et al. (2018) found that rs619586 AG and GG genotypes in MALAT1 are associated with reduced risk of coronary atherosclerotic heart disease in a Chinese population and play protective roles in preventing the occurrence of coronary atherosclerotic heart disease. A study by Peng et al. (2018) found that the lncRNA MALAT1 rs619586 AG genotype and the rs3200401 CT genotype are associated with a decreased susceptibility to breast cancer, and compared to the rs619586AA genotype, carriers with the rs619586 G variant have lower expression of MALAT1 in the Chinese Han population. Similarly, in our case-control study, the results suggested that the MALAT1 gene rs619586 G variant decreased the risk of recurrent miscarriage in a southern Chinese population and that it was likewise a protective factor against recurrent miscarriage susceptibility. Although we did not detect the expression of MALAT1 in miscarriage patients, we speculate that the rs619586 G variant may reduce the risk of miscarriage by regulating the expression of MALAT1. In future research, we will detect the expression level of MALAT1 and further verify our speculation with larger sample sizes. MALAT1 rs591291 showed significantly better hepatocellular cancer prognoses in female patients (Wang B.G. et al., 2018). Another study by Zhu et al. (2018) found that MALAT1 gene polymorphisms (rs619586 and rs3200401) were not significantly associated with ischemic stroke susceptibility in a northern Chinese Han population. However, these studies suggest that MALAT1 gene polymorphisms may play different roles in different diseases. In our case-control study, two SNPs of the MALAT1 gene (rs3200401 and rs591291) were not related to recurrent miscarriage susceptibility. These results suggest that MALAT1 gene variants may play similar roles in the pathological processes of recurrent miscarriage and cardiovascular diseases. To the best of our knowledge, this case-control study is the first to validate the association between genetic variants of lncRNA MALAT1 (rs619586, rs3200401, and rs591291) and recurrent miscarriage susceptibility. We propose that the rs619586 G variant may play a significant role in the pathogenesis of recurrent miscarriage. Dysregulation of MALAT1 contributes to various human diseases. MALAT1 is upregulated in many types of cancer, myocardial infarction, diabetes mellitus, and diabetic retinopathy. MALAT1 mainly regulates inflammation, cell proliferation, migration, and metastasis and affects endothelial function (Zhang et al., 2017; Masoumi et al., 2018). Cardiovascular diseases and diabetes mellitus are high risk factors for miscarriage. The rs619586 G variant may reduce the risk of miscarriage by regulating the expression of MALAT1. Currently, the molecular mechanism of MALAT1 in miscarriage patients is still not clear. Therefore, further studies of the functional role of MALAT1 in miscarriage are needed.

Numerous studies have demonstrated that advanced age is a risk factor for miscarriage; beyond the age of 40 years, the risk of miscarriage in women is five times greater than that in 31- to 35-year-old women (van Kooij et al., 1996; Nybo Andersen et al., 2000; Agenor and Bhattacharya, 2015). This study also confirmed that the number of prior miscarriages is strongly associated with the risk of miscarriage, consistent with previous reports that miscarriage rates increase with the number of previous miscarriages (Ogasawara et al., 2000). Kharazmi et al. (2010) found that women with a history of miscarriage have increased risk of myocardial infarction. However, some previous research findings have suggested that the rs619586 G variant is associated with decreased risk of coronary atherosclerotic heart disease and congenital heart disease (Li et al., 2018; Wang G. et al., 2018). Similarly, we found that the rs619586 AG/GG variant was more protective in women less than 35 years of age and in women with two to three miscarriages than the rs619586 AA variant. Moreover, combined analysis suggested that the presence of two or three protective genotypes decreased the recurrent miscarriage risk in women less than 35 years of age and in women who had undergone 2–3 miscarriages, which may be one reason that the incidence of recurrent miscarriage was relatively low in women less than 35 years of age. The molecular mechanism underlying this phenomenon deserves further exploration. In addition, further studies with larger sample sizes are needed to confirm these results.

This case-control study was the first to evaluate the association between lncRNA MALAT1 polymorphisms and recurrent miscarriage susceptibility. However, several limitations of our research should be noted. First, we only studied the relationships between lncRNA MALAT1 gene polymorphisms and susceptibility to recurrent miscarriage; MALAT1 gene expression in patients was not determined. Second, only three SNPs (rs619586, rs3200401, and rs591291) were analyzed in our study. Other SNPs, such as rs11227209, should be included in future research. Third, in the stratified analysis, we only analyzed the association of age and number of miscarriages with lncRNA MALAT1 gene polymorphisms. Because our study is retrospective, we were not able to collect and control for other factors, such as smoking, drinking status, eating habits, and these are important factors in miscarriage. Fourth, the sample size of this study, with 640 participants, was still not sufficiently large, which may have limited the statistical power. Future studies with larger sample sizes and inclusion of other factors that are important for miscarriage are needed to validate our findings regarding the roles of the lncRNA MALAT1 gene in recurrent miscarriage susceptibility.

In summary, our study confirmed the significant protective effect of the MALAT1 rs619586 G variant in recurrent miscarriage in a Chinese population. Moreover, the protective effect was more pronounced in women less than 35 years of age, than in women of other age groups; in addition, the protective effect was stronger in subgroups of women who had undergone two to three miscarriages, than in other subgroups. Thus, the rs619586 G allele may be involved in decreasing the number of miscarriages. However, future studies with larger sample sizes and practical experiments should be performed to further validate the roles of MALAT1 gene variants in recurrent miscarriage susceptibility.

## Ethics Statement

This study was approved by the Ethics Committee of Guangzhou Women and Children’s Medical Center (Guangzhou, China). Written informed consent was obtained from each recurrent miscarriage patient and control subject before participation in the study.

## Author Contributions

All authors contributed significantly to this work and supported the publication of the manuscript. DC, LL, and ZF devised the research plan. ZF and QL analyzed the data. DC wrote the manuscript. YT, YY, ZL, and HZ performed the experiments. LP and LF designed the experimental methods. XG and QX modified and polished the manuscript.

## Conflict of Interest Statement

The authors declare that the research was conducted in the absence of any commercial or financial relationships that could be construed as a potential conflict of interest.
